# GhWRKY70D13 Regulates Resistance to *Verticillium dahliae* in Cotton Through the Ethylene and Jasmonic Acid Signaling Pathways

**DOI:** 10.3389/fpls.2020.00069

**Published:** 2020-02-25

**Authors:** Xian-Peng Xiong, Shi-Chao Sun, Xin-Yu Zhang, Yan-Jun Li, Feng Liu, Qian-Hao Zhu, Fei Xue, Jie Sun

**Affiliations:** ^1^ Key Laboratory of Oasis Eco-Agriculture, College of Agriculture, Shihezi University, Shihezi, China; ^2^ Agriculture and Food, CSIRO, Canberra, ACT, Australia

**Keywords:** *Gossypium hirsutum*, *Verticillium dahliae*, *GhWRKY70D13*, ribonucleic acid interference, jasmonic acid, ethylene

## Abstract

*Verticillium* wilt caused by *Verticillium dahliae* is a destructive cotton disease causing severe yield and quality losses worldwide. WRKY transcription factors play important roles in plant defense against pathogen infection. However, little has been reported on the functions of WRKYs in cotton's resistance to *V. dahliae*. Here, we identified 5, 5, and 10 *WRKY70* genes in *Gossypium arboreum*, *Gossypium raimondii*, and *Gossypium hirsutum*, respectively, and investigated the expression profiles of all *GhWRKY70* genes in various cotton tissues and in response to hormone treatment or *V. dahliae* infection. Reverse transcription-quantitative PCR analysis showed that *GhWRKY70D13* was expressed higher in roots and stems than in other tissues, and up-regulated after *V. dahliae* inoculation. Knock-down of *GhWRKY70D13* improved resistance to *V. dahliae* in both resistant and susceptible cotton cultivars. Comparative analysis of transcriptomes generated from wild-type and stable RNAi (RNA interference) plant with down-regulated *GhWRKY70D13* showed that genes involved in ethylene (ET) and jasmonic acid (JA) biosynthesis and signaling were significantly upregulated in the *GhWRKY70D13* RNAi plants. Consistently, the contents of 1-aminocyclopropane-1-carboxylic (ACC), JA, and JA-isoleucine levels were significantly higher in the *GhWRKY70D13* RNAi plants than in wild-type. Following *V. dahliae* infection, the levels of ACC and JA decreased in the *GhWRKY70D13* RNAi plants but still significantly higher (for ACC) than that in wild-type or at the same level (for JA) as in non-infected wild-type plants. Collectively, our results suggested that *GhWRKY70D13* negatively regulates cotton's resistance to *V. dahliae* mainly through its effect on ET and JA biosynthesis and signaling pathways.

## Introduction

Cotton (*Gossypium hirsutum*) produces important natural fibers and is one of the most important industrial crops globally. Cotton production is constantly threated by various biotic and abiotic stresses, such as cold, drought, insect pests, and pathogens. The soil-borne fungus *Verticillium dahliae* is one of the most destructive pathogens in cotton production areas worldwide (Cai et al., 2009; Wang et al., 2016; Gong et al., 2017). *Verticillium* wilt caused by *V. dahliae* is difficult to control since *V. dahliae* has a wide host range and its microsclerotia can survive for long periods in the soil even in the absence of suitable hosts (Fradin and Thomma, 2006; Shaban et al., 2018). Moreover, there is no effective fungicide that can prevent cotton from *V. dahliae* infection (Mol et al., 1995; Wang et al., 2016).

The most economical and efficient method to control *V. dahliae* is to improve host resistance. However, no germplasms of *G. hirsutum* cultivars have shown immune to *Verticillium* wilt (Yang et al., 2015). Thus, it is essential to identify *Verticillium* wilt resistance genes in cotton germplasm and incorporate them into elite cotton cultivars. In general, the *V. dahliae* strains are classified as defoliating or nondefoliating which have different pathogenic characteristics (Chen et al., 2018; Zhang et al., 2019). Although numerous *V. dahliae*-resistance genes have been characterized in cotton, including *GbWRKY1* (Li et al., 2014a), *GhCYP94C1* (Sun et al., 2014), *GhERF6* (Yang et al., 2015), *GhHDTF1* (Gao et al., 2016), *GhERF1-like* (Guo et al., 2016), *GhbHLH171* (He et al., 2018), and *GhJAZ2* (He et al., 2018), only a defoliating strain of *V. dahliae* was used in most of these studies. There is still limited knowledge of the genetic and molecular mechanisms on the interaction between cotton and *V. dahliae*.

Plant hormones, such as jasmonic acid (JA) and ethylene (ET), are crucial for plant defenses against pathogen infection. JA signaling plays a role in the resistance against necrotrophic pathogens (Glazebrook, 2005). JA has been found to induce degradation of Jasmonate ZIM domain (JAZ) proteins and to de-repress the MYC2 transcription factors to activate JA responsive gene expression (Chini et al., 2007; Chung and Howe, 2009; Zhang et al., 2015a). JA-related genes have a positive role in the defense of plant against *V. dahliae* in *Arabidopsis thaliana* and cotton (Gao et al., 2013; Li et al., 2014a; He et al., 2018). ET often synergizes with JA and increases plant tolerance to necrotrophic pathogens (Zhu et al., 2011). ET is a small gaseous hormone involved in plant innate immunity against pathogens, and various studies have revealed that ET plays a role in resistance to *V. dahliae* in tobacco and cotton (Yang et al., 2015; Guo et al., 2016; Liu et al., 2017).

Many transcription factors have important regulatory functions in plant defenses against pathogens (Nuruzzaman et al., 2013; Jiang et al., 2017). As stress-responsive transcription factors, plant WRKY proteins play important roles in plant immunity (Rushton et al., 2010; Jiang et al., 2017). The WRKY family belongs to one of the largest families of transcriptional regulators in plants and is characterized by a highly conserved WRKY domain (about 60 amino acids) at the N-terminus and an atypical zinc finger structure at the C-terminus (Rushton et al., 2010). Based on the number of WRKY domains and the structure of their zinc fingers, 74 WRKY transcription factors were identified and divided into three groups in *Arabidopsis* (Eulgem et al., 2000). The group III WRKY transcription factors are involved in plant resistance to pathogens (Li et al., 2004; Li et al., 2006; Murray et al., 2007; Higashi et al., 2008; Kim et al., 2008; Hu et al., 2012). In *Arabidopsis*, *wrky53, wrky38,* and *wrky62* mutant plants displayed severer susceptibility to *Pseudomonas syringae* pv. *tomato* DC3000 (*Pst* DC3000) (Murray et al., 2007; Kim et al., 2008; Hu et al., 2012), and overexpression of *AtWRKY41* enhances disease resistance to *Pst* DC3000 (Higashi et al., 2008). Similarly, overexpression of *AtWRKY70* improves resistance to necrotrophic pathogens by activating the salicylic acid (SA) signaling pathway and suppressing the JA signaling pathway (Li et al., 2004; Li et al., 2006).

Recently, genome-wide identifications of the WRKY family genes have been carried out in *Gossypium raimondii*, *Gossypium arboreum*, and *G. hirsutum* (Dou et al., 2014; Ding et al., 2015), but only few studies have reported WRKY-regulated responses to *V. dahliae* infection in cotton (Li et al., 2014a; Xiong et al., 2019). Therefore, research to understand the biological functions and mechanisms of WRKY genes in resistance against *V. dahliae* in cotton remains an important goal. Our previous study showed that *GhWRKY70A05a* acted as a negative regulator in cotton's resistance against *V. dahliae* by repressing JA signaling pathway and promoting SA signaling pathway (Xiong et al., 2019). In order to get broader insight into the roles of *GhWRKY70* genes in defense against *V. dahliae*, we investigated the expression of 10 *GhWRKY70* genes in *G. hirsutum* in response to *V. dahliae* infection and to treatment with the plant hormones SA, methyl jasmonate (MeJA), and ethephon (ETH). We found that *GhWRKY70D13* was highly expressed in both roots and stems, and was induced in roots of both *V. dahliae*-susceptible and resistant cotton cultivars upon *V. dahliae* infection. Using the VIGS and RNA interference (RNAi) approaches, we demonstrated that silencing the expression of *GhWRKY70D13* enhanced cotton's resistance to *V. dahliae*, which was likely achieved through upregulating the JA and/or ET biosynthesis and signaling pathways, a potential mechanism different from that of *GhWRKY70A05a*.

## Materials and Methods

### Plant Materials

*V. dahliae*-resistant *G. hirsutum* cv. Zhongzhimian 2 and *V. dahliae*-susceptible *G. hirsutum* cv. Xinluzao 7 were used for gene expression analysis under *V. dahliae*, SA, MeJA, or ETH treatment. Zhongzhimian 2, Xinluzao 7 and another *V. dahliae*-susceptible *G. hirsutum* cv. Xincai 7 were used in virus-induced gene silencing (VIGS) experiments and disease assays. All plants were grown in a greenhouse with a 16-h-light/8-h-dark cycle at 24/23°C (light/dark), watered with Hoagland's nutrient solution weekly.

### Identification of the *GhWRKY70* Gene Family in Cotton

The protein sequences of *Arabidopsis* group III WRKYs were obtained from the The Arabidopsis Information Resource (TAIR) website (https://www.arabidopsis.org/). The AtWRKY70 protein sequence was used as a query to search the genome sequences of *G. raimondii*, *G. arboreum*, and *G. hirsutum*, which were retrieved from Cottongen (https://www.cottongen.org/), with an E-value less than 1 x 10^−30^. To identify *WRKY70* genes in the three cotton genomes, the hits were further filtered using the Pfam database (http://pfam.xfam.org) and the National Center for Biotechnology Information (NCBI) Conserved Domain Database (http://www.ncbi.nlm.nih.gov/Structure/cdd/wrpsb.cgi) based on the presence of the WRKY domain and the C2HC zinc finger.

### Gene Cloning, Multiple-Sequence Alignment, and Phylogenetic Analysis

The coding sequences of *GhWRKY70A06* and *GhWRKY70D13* were amplified from roots of Zhongzhimian 2 using gene specific primers (**Supplementary Table S4**). The amplified products were cloned into the pMDT-19 vector, and confirmed by sequencing (Sangon Biotech Co., Ltd., Shanghai, China). *GhWRKY70A05a* has been previously cloned (Xiong et al., 2019). The ClustalX software (ver. 1.83) was employed for alignment of the WRKY70 amino acid sequences from the three cotton species and the group III *WRKY70* genes from *Arabidopsis*. The phylogenetic tree was constructed using the neighbor-joining method implemented in the Molecular Evolutionary Genetics Analysis (MEGA) software 7.1 with a bootstrap value of 1,000.

### Analysis of Cis-Acting Elements in the Promoters of *GhWRKY70* Genes

The 2-kb genomic sequences upstream the transcriptional start sites of *GhWRKY70* genes were downloaded from Cottongen (https://www.cottongen.org/) and used in the analysis of cis-acting elements using the PlantCARE database.

### Application of Plant Hormones

Two-leaf stage cotton seedlings were sprayed with 1 mmol L^−1^ SA, 100 μmol L^−1^ MeJA, or 5 mg L^−1^ ETH. Cotton seedlings sprayed with distilled water were used as controls. Roots were collected from the treated seedlings at 0, 0.5, 1, 3, 6, 9, 12, and 24 h after treatment, frozen immediately in liquid nitrogen and then stored at −80°C for future use. All treatments were done with three independent biological replicates.

### Inoculations With *Verticillium dahliae*

A highly aggressive defoliating strain, V991, was kindly provided by Professor Longfu Zhu (Huazhong Agricultural University) and used in a disease assay. *V. dahliae* inoculation was performed according to a previously described method (Xiong et al., 2019). Two-leaf stage cotton plants were infected using 10 ml *V. dahliae* spore suspension (10^6^ spores per ml) by irrigating injured roots. A similar routine was adopted for treating the control plants with distilled water. Cotton roots from five individual seedlings were harvested for RNA extraction at 0, 1, 3, 6, 12, 24, 48, and 72 h post-inoculation (hpi). Samples of three biological replicates were prepared. Disease index at 14 and 21 day post-inoculation (dpi) was calculated based on 30 VIGS or *GhWRKY70D13* RNAi transgenic cotton plants. The fungal recovery assay and calculations of relative fungal biomass were performed as previously described (Ellendorff et al., 2009).

### Ribonucleic Acid Isolation and Expression Pattern Analysis

Total RNA was extracted from cotton tissues with an RN09-EASYspin RNA Plant Mini Kit (Aidlab Biotechnologies Co. Ltd., Beijing, China) according to the manufacturer's instructions. All RNA samples were treated with DNase I (Takara Biotechnology Co., Ltd., Dalian, China) to remove genomic DNA. RNA quantity was determined using a NanoDrop 2000C Spectrophotometer (Thermo Scientific, Wilmington, USA). First-strand complementary DNA (cDNA) was synthesized using the PrimeScript II 1st Strand cDNA Synthesis Kit (Takara). The gene-specific primers used in reverse transcription-quantitative PCR (RT-qPCR) were designed using qPrimerDB (https://biodb.swu.edu.cn/qprimerdb/). The lengths of the amplified fragment were designed to be between 80 and 250 bp. qPCR reactions were performed on a Light Cycler 480II (Roche, Rotkreuz, Switzerland) sequence detection system using SYBR Green (Roche) including a pre-incubation at 95°C for 10 min, followed by 40 cycles of denaturation at 95°C for 15 s, annealing at 60°C for 15 s, and extension at 72°C for 15 s. The cotton *UBQ7* (DQ116441) gene was used as an internal control. Each experiment included three biological replicates. The primers used in RT-qPCR are listed in **Supplementary Table S4**. The expression data of the 10 *GhWRKY70* genes in other tissues were based on previously published transcriptomic data from TM-1 (Zhang et al., 2015b).

### Virus-Induced Gene Silencing Assays

The vectors of tobacco rattle virus (TRV) were used for VIGS to investigate the function of *GhWRKY70* in response to *V. dahliae* infection (Liu et al., 2002). The *TRV*: *GhCHLI* (encoding magnesium chelatase subunit I) was used as a positive control for the VIGS efficiency (Gu et al., 2014), because leaves of the *TRV*: *GhCHLI* plants would show a bleaching phenotype. Approximately 400 bp gene-specific fragment from the coding sequence of *GhWRKY70D04*, *GhWRKY70D13,* or *GhCHLI* was amplified from *G. hirsutum* cv. Zhongzhimian 2 and inserted into the *TRV2* vector, respectively. The primers used in construction of the VIGS vectors are listed in **Supplementary Table S4**. *TRV*: *GhWRKY70D04*, *TRV*: *GhWRKY70D13*, *TRV*: *GhCHLI* or *TRV1* construct was transformed into *Agrobacterium tumefaciens* strain GV3101 by electroporation. We then followed the same procedures as previously reported to carry out VIGS in cotton (Gao et al., 2013). RNA was extracted from the *TRV: GhWRKY70D13* cotton leaves to measure the expression level of *GhWRKY70*.

### Ribonucleic Acid Interference Construct and Cotton Transformation

The construct used in generating *GhWRKY70D13* RNAi transgenics was generated by insert an amplified *GhWRKY70D13* fragment (324-bp) into the PANDA35 HK vector through a recombination reaction. The RNAi vector was transformed into *G. hirsutum* cv. Xincai 7 *via A. tumefaciens* (LBA4404) as previously described (Jin et al., 2006). The regenerated plants were examined by antibiotic kanamycin and RT-qPCR to verify the presence and expression of the transgene. T_3_ homozygous lines were identified and used in all experiments.

### Transcriptome Analysis

RNA-seq was used to investigate the effect of knocking-down of *GhWRKY70D13* expression on disease responses. Root samples were collected from wild-type (WT) and the two *GhWRKY70D13-*RNAi lines (Ci1 and Ci2) at 0, 24, and 72 hpi following *V. dahliae* inoculation. The samples from Ci1 and Ci2 were mixed to form a single sample (designed Ci) based on time points and biological replicates. Finally, 18 samples (2 genotypes x 3 time points x 3 biological replicates) were used in transcriptome sequencing. Total RNA was extracted with a Tiangen RNA extraction kit (Tiangen, Beijing, China) according to the manufacturer's instructions. RNA purity and integrity were checked before library construction, using NanoPhotometer^®^ spectrophotometer (Implen, CA, USA) and the Bioanalyzer 2100 System (Agilent Technologies, CA, USA), respectively. Approximately 3 μg RNA per sample was used for library construction. RNA sequencing was performed by Novogene (Novogene, Tianjin, China) with an Illumina HiSeq (Illumina, CA, USA) system. The RNA-seq raw data were checked for quality. After removing the low quality reads, the remaining clean reads were aligned to the *G. hirsutum* genome using TopHat v2.0.12 (Wang et al., 2019). The raw data of transcriptome sequencing were deposited in NCBI under PRJNA578842.

### Identification and Functional Annotation of Differentially Expressed Genes

Gene expression levels were determined by FPKM (fragments per kilobase of transcript sequence per millions of base pairs sequenced) (Trapnell et al., 2010). Using DESeq, the transcripts with an adjusted *P*-value < 0.05 were considered to be differentially expressed genes (DEGs) (Anders and Huber, 2010). The DEGs were subjected to KEGG (Kyoto Encyclopedia of Genes and Genomes) pathway analysis to identify statistically enriched pathways (*P*-value < 0.05) (Kanehisa et al., 2008). Venn diagrams and heat-maps were generated using the tools available on the Novemagic server (https://magic.novogene.com).

### Quantification of the Contents of 1-Aminocyclopropane-1-Carboxylic, Jasmonic Acid, Jasmonoyl Isoleucine, and Salicylic Acid

WT, Ci1 (*GhWRKY70D13-*RNAi line 1), and Ci2 (*GhWRKY70D13-*RNAi line 2) seedlings at the two-leaf stage were inoculated with *V. dahliae* through the root irrigation method. Fresh root samples of WT, Ci1, and Ci2 from three biological replicates were harvested at 0 and 72 hpi. Quantification of the content of each phytohormone was performed by Jiangxi Likon Science & Technology Co., Ltd (Nanchang, China). Extraction and measurement of endogenous ACC were performed as previously described (Datta et al., 2015).

The levels of JA, JA-isoleucine (JA-Ile), and SA were determined using a previously described method with slight modification (Sun et al., 2014). Approximately 0.2 g fresh samples were ground to a fine powder in liquid nitrogen and mixed with 2 ml pre-cooled extraction buffer (methanol:water, 80:20, v:v). After shaking at 4°C for 16 h in the dark, the samples were centrifuged at 12,000 rpm for 10 min at the same temperature with 0.3 ng added SA (Sigma) as an internal standard. The supernatant was dried under N2 at room temperature, then dissolved in 0.4 ml methanol and filtered with a 0.22 mm filter membrane. The JA and JA-Ile levels were quantified using the high-performance liquid chromatography-tandem mass spectrometry (HPLC-MS/MS) system (AB SCIEX Triple Quad 5500) with JA (Sigma) and JA-Ile (Sigma) as the external standards.

## Results

### Identification of WRKY70 Transcription Factors in Cotton

Using AtWRKY70 as a query, we identified 5, 5, and 10 WRKY70 transcription factors in *G. raimondii* (Wang et al., 2012), *G. arboreum* (Li et al., 2014b), and *G. hirsutum* (Zhang et al., 2015b; Wang et al., 2019), respectively, indicating no gene loss occurred after polyploidization. They were named based on their chromosomal location (**Supplementary Tables S1** and **S2**). Apart from *GhWRKY70A05a*, *GhWRKY70A06*, and *GhWRKY70D13*, other *GhWRKY70* genes were annotated consistently in the two TM-1 genomes (*G. hirsutum*) (Zhang et al., 2015b; Wang et al., 2019) (**Supplementary Table S2**). To reconcile the annotation difference of *GhWRKY70A05a*, *GhWRKY70A06*, and *GhWRKY70D13*, we cloned their coding sequences (**Supplementary Table S3**), and confirmed one of the two annotations was correct for each gene (**Supplementary Table S2**).

### Conserved Motifs, Phylogenetic and Cis-Acting Element Analysis of the *GhWRKY70* Genes

Amino acid sequence alignment was performed using all the *GhWRKY70* genes identified in *G. raimondii*, *G. arboreum*, and *G. hirsutum* together with *AtWRKY70*. Conserved domain analysis confirmed that all cotton WRKY70s identified belong to the group III with a well-conserved WRKY domain at the N-terminal region and a C2HC zinc finger at the C-terminus (**Supplementary Figure S1A**). The phylogenetic relationships among cotton WRKY70s and *Arabidopsis* group III WRKY proteins were further analyzed through construction of a phylogenetic tree using the maximum likelihood method. It was revealed that each pair of homeologs from the At and Dt subgenomes of *G. hirsutum* was unambiguously grouped with their orthologs in the diploids *G. raimondii* and *G. arboreum* (**Supplementary Figure S1B**), suggesting the conservation of each gene during the history of cotton evolution. *GhWRKY70A02*, *GhWRKY70A05a*, *GhWRKY70A05b*, *GhWRKY70A06*, and *GhWRKY70A13* of At homologs were corresponding to *GhWRKY70D02*, *GhWRKY70D05a*, *GhWRKY70D04*, *GhWRKY70D06*, and *GhWRKY70D13* of Dt homologs, respectively.

To identify the putative cis-acting elements in the promoters of *GhWRKY70* genes, a 2-kb sequence upstream of the start codon of each *GhWRKY70* was scanned using the online tool PlantCARE. The results showed that each *GhWRKY70* contained at least one hormone responsive cis-element in its promoter (**Supplementary Figure S2**). Nine of the 10 *GhWRKY70* genes contained SA (TCA-element) and MeJA responsive elements (TGACG-element) in their promoters. Eight contained the ET-responsive element (ERE). Five contained the gibberellin responsive element (GARE-motif) and abscisic acid responsive element (ABRE). The auxin-responsive element (TGA-element) was identified in three *GhWRKY70* genes. *GhWRKY70D05a* contained cis-elements responsive to all hormones in its promoter. These data suggested that most *GhWRKY70* genes might be regulated by one or more hormones, and could play important roles in hormonal responses.

### Expression Patterns of *GhWRKY70* Genes in Different Tissues

**Figure 1:**
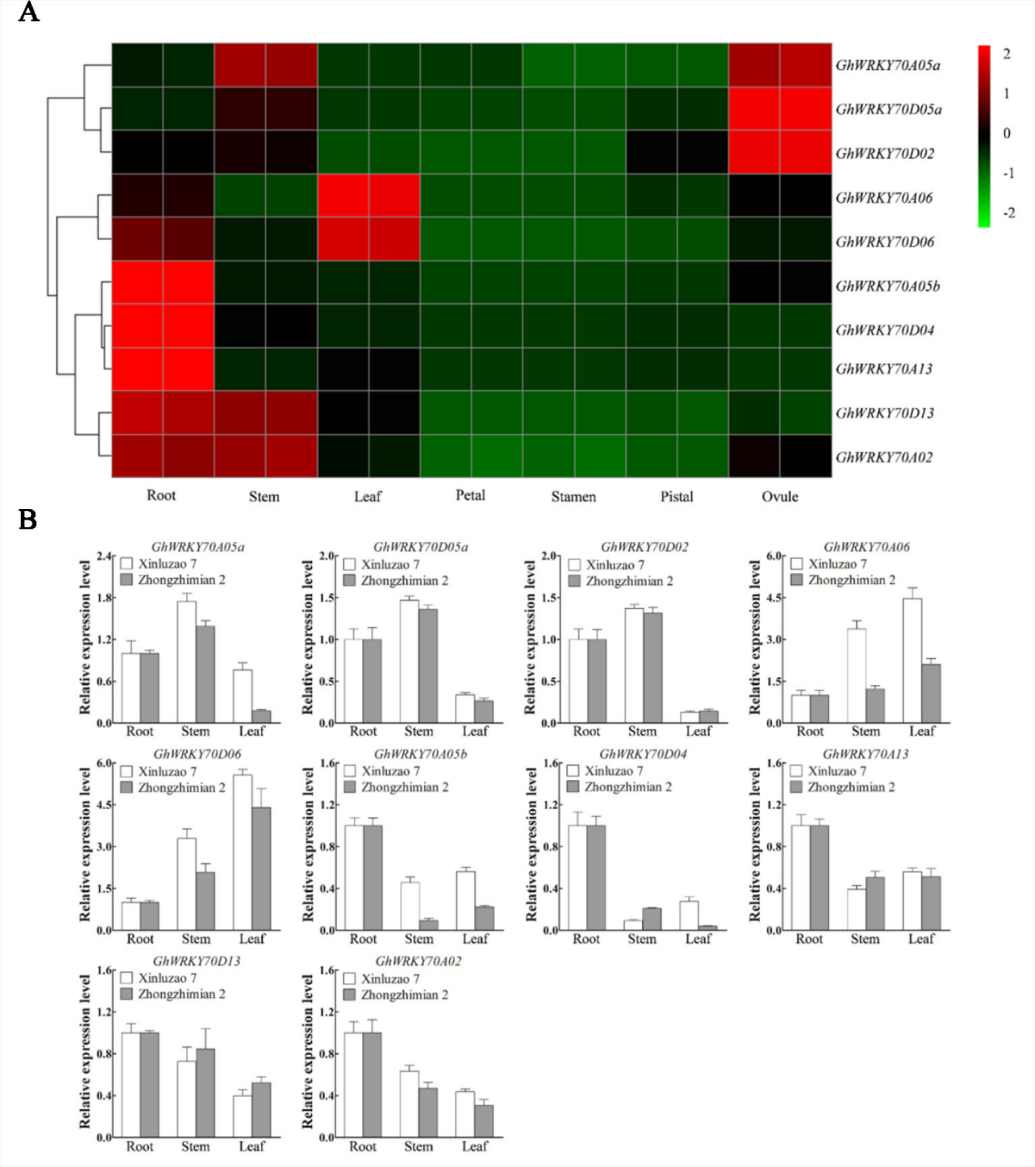
Expression patterns of *GhWRKY70* genes in different cotton tissues. **(A)** Heat map showing the expression level of the 10 *GhWRKY70* genes in various tissues (root, stem, leaf, petal, stamen, pistil, and ovule) of TM-1 based on RNA-seq data. **(B)** Expression profiling of *GhWRKY70* genes in various tissues based on reverse transcription-quantitative PCR. Two-leaf stage cotton seedlings were used in the analyses. For both cultivars Xinluzao 7 and Zhongzhimian 2, root was used as the reference sample and its expression level was set to 1.0. Cotton *GhUBQ7* was used as the internal control to normalize all data. Each experiment was performed using three independent biological replicates.

To investigate the expression patterns of the *GhWRKY70* genes in different tissues, we used published RNA-seq data [*G. hirsutum* cv. TM-1; (Zhang et al., 2015b)] (**Figure 1A**), and did qPCR to quantify their expression levels in roots, stems, and leaves of Zhongzhimian 2 (*V. dahliae* resistant) and Xinluzao 7 (*V. dahliae* susceptible) (**Figure 1B**). The two observations were the tissue-specificity of individual genes and the similar expression profile of the two homeologs of most pairs of *GhWRKY70* from the At and Dt subgenomes. Generally, the *GhWRKY70* genes, except *GhWRKY70D02* and *GhWRKY70D05a*, had a expression level higher in vegetative tissues than in reproductive tissues. Both *GhWRKY70A06* and *GhWRKY70D06* had higher expression levels in leaves than in other tissues (**Figure 1A**). *GhWRKY70A05b*, *GhWRKY70D04*, and *GhWRKY70A13* had a higher expression level in roots than in other tissues. *GhWRKY70D13* and *GhWRKY70A02* exhibited higher expression levels in both roots and stems, while *GhWRKY70A05a* exhibited a higher expression level in stems and ovules (**Figure 1A**). Similar expression patterns of the two homologs suggest their possible redundant functions. The qPCR results were consistent with the RNA-seq results and some *GhWRKY70* genes showed different expression profiles in the two *G. hirsutum* cultivars with different *V. dahliae* resistance (**Figure 1B**).

### Expression Analysis of the *GhWRKY70* Genes in Response to Hormone Treatments and *Verticillium dahliae* Inoculation

**Figure 2:**
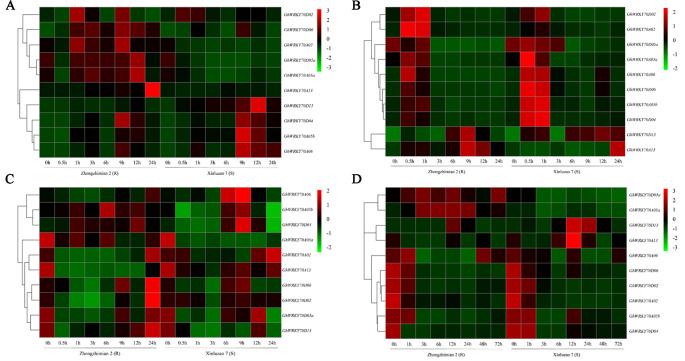
Expression patterns of the 10 *GhWRKY70* genes from cotton under different hormone treatments and after *Verticillium dahliae* inoculation. **(A–C)** Expression changes of the 10 *GhWRKY70* genes in roots of Zhongzhimian 2 (*V. dahliae*-resistant) and Xinluzao 7 (*V. dahliae*-susceptible) after treatment with **(A)** SA, **(B)** MeJA, and **(C)** ETH. Samples collected at 0, 0.5, 1, 3, 6, 9, 12, and 24 h after hormone treatment were used in real-time (RT)-qPCR. **(D)** Expression changes of the 10 *GhWRKY70* genes in roots of Zhongzhimian 2 (*V. dahliae*-resistant) and Xinluzao 7 (*V. dahliae*-susceptible) after inoculation with *V. dahliae* strain Vd991. Samples collected at 0, 1, 3, 6, 12, 24, 48, and 72 h after inoculation were used in reverse transcription-quantitative PCR. Two-leaf stage cotton seedlings were used in the treatment of hormones or *V. dahliae*. Each experiment was performed using three independent biological replicates.

Analysis of hormone responsive elements in the promoters of *GhWRKY70* genes suggests their expression might be induced by SA, MeJA, and/or ET. We thus investigated the effects of SA, MeJA, and ET on the changes of the transcript level of the 10 *GhWRKY70* genes in Zhongzhimian 2 (resistant to *V. dahliae*) and Xinluzao 7 (susceptible to *V. dahliae*) using RT-qPCR. Upon SA treatment, the 10 *GhWRKY70* genes were clustered into two groups. One group showed upregulation in Zhongzhimian 2 but not in Xinluzao 7, while another group showed upregulation in both cultivars (**Figure 2A**). *GhWRKY70A02*, *GhWRKY70A05a*, and *GhWRKY70D05a* were significantly (*P* < 0.01) downregulated by 3.0- to 87.4-fold, 1.4- to 5.9-fold, and 2.2- to 8.1-fold in the *V. dahliae* susceptible cultivar and upregulated by 1.5- to 2.7-fold, 1.6- to 2.1-fold, and 1.4- to 2.2-fold in the *V. dahliae* resistant cultivar, respectively (**Figure 2A**). In response to SA treatment, the expression level of *GhWRKY70D13* increased gradually from 0.5 to 12 h, while significant (*P* < 0.01) upregulation of *GhWRKY70A13* was observed at 24 h by 17.5-fold in the *V. dahliae* resistant cultivar (**Figure 2A**).

In response to MeJA treatment, the 10 *GhWRKY70* genes generally also showed two patterns, one showing significant (*P* < 0.01) upregulation at 0.5 h and/or 1 h, and then downregulation afterwords in both cultivars, another (including *GhWRKY70A13* and *GhWRKY70D13*) showing upregulation at 6–24 h, particularly *GhWRKY70D13* increasing 7.6- to 38.3-fold and 23.2- to 29.1-fold at 6-24 h following MeJA treatment in Zhongzhimian 2 and Xinluzao 7, respectively (**Figure 2B**).

In response to ETH treatment, six *GhWRKY70* genes in Zhongzhimian 2 and Xinluzao 7 were significantly (*P* < 0.01) suppressed at 0.5-24 h by 0.32- to 0.95-fold (**Figure 2C**). ETH treatment seemed to induce the expression of *GhWRKY70A05a* and *GhWRKY70A06*. In Xinluzao 7, *GhWRKY70A05b* and *GhWRKY70D04* were significantly (*P* < 0.01) upregulated by 1.7- and 2.4-fold at 9 h. *GhWRKY70D13* was downregulated by 1.5- to 2.3-fold from 0.5 to 9 h but restored its expression at 12 h in Zhongzhimian 2 (**Figure 2C**).

In response to *V. dahliae* infection, the expression patterns of the 10 *GhWRKY70* genes could be divided into three groups (**Figure 2D**). Group I contains *GhWRKY70A05a* and *GhWRKY70D05a*. Both genes exhibited significant upregulation and downregulation in the *V. dahliae*-resistant and -susceptible cultivar, respectively. Group II includes *GhWRKY70D13* and *GhWRKY70A13*, that were induced by 11.1- and 3.1-fold at 12 hpi in *V. dahliae*-susceptible cultivar Xinluzao 7 following *V. dahliae* infection. However, in *V. dahliae*-resistant cultivar Zhongzhimian 2, upregulation was only observed in *GhWRKY70D13* (6.6-fold) but not in *GhWRKY70A13* at 12 hpi. The remaining six genes belongs to group III. They were generally downregulated in response to *V. dahliae* infection (**Figure 2D**). These results suggest that the 10 *GhWRKY70* genes may have different functions in defense against *V. dahliae* infection in cotton. Due to its consistent upregulation in both *V. dahliae* resistant and susceptible cultivars upon *V. dahliae* infection, *GhWRKY70D13,* together with a representative (*GhWRKY70D04*) from group III, was chosen for further functional characterization.

### Silencing of *GhWRKY70D13* Improves Resistance to *Verticillium dahliae* in Cotton

**Figure 3:**
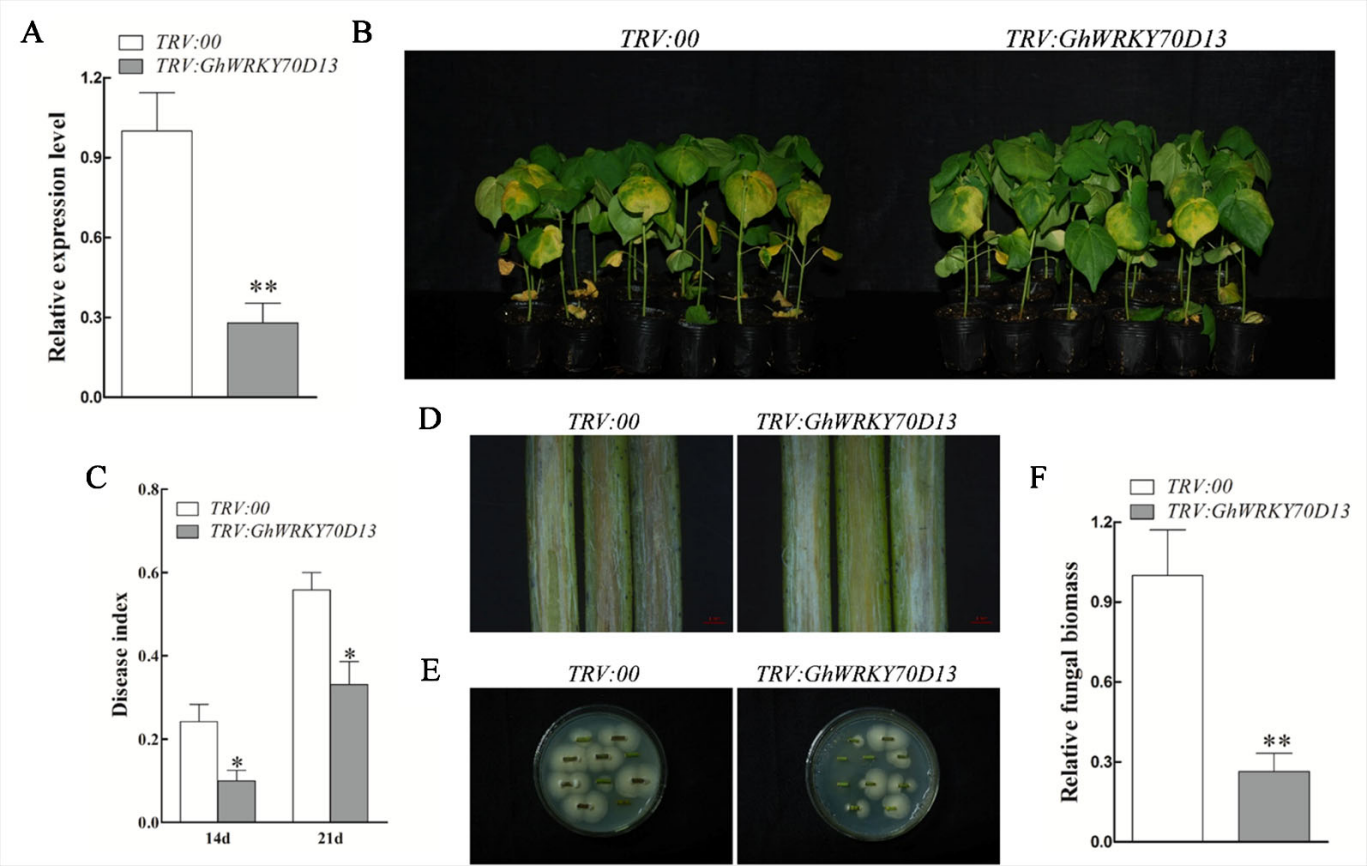
Silencing of *GhWRKY70D13* enhanced resistance to *Verticillium dahliae* in *V. dahliae*-susceptible cotton cultivar Xincai 7. **(A)** The expression level of *GhWRKY70D13* in the *TRV:00* and *TRV: GhWRKY70D13* plants. Total RNA was isolated from leaves at 10 days post-agroinfiltration. *GhUBQ7* was used as the control. Each experiment was performed using three independent biological replicates. **(B)** Disease symptoms of the *TRV:00* and *TRV: GhWRKY70D13* plants after *V. dahliae* inoculation. Photographs were taken at 14 days after inoculation with 30 plants per treatment. **(C)** Disease index of the *TRV:00* and *TRV: GhWRKY70D13* plants at 14 days and 21 days after inoculation with *V. dahliae*. Each experiment was performed using three independent biological replicates. **(D)** Disease symptoms in stems of the *TRV:00* and *TRV: GhWRKY70D13* plants at 14 days after inoculation with *V. dahliae*. Vascular browning was observed in the *TRV:00* plants but not in the *TRV: GhWRKY70D13* plants. **(E)** Comparison of fungal growth in stem sections prepared from the *TRV:00* and *TRV: GhWRKY70D13* plants at 14 days after *V. dahliae* inoculation. PDA medium was used in the culture of stems. Photographs were taken after 7 days of culture at 25°C. **(F)** qPCR analysis of the relative fungal biomass in stems of the *TRV:00* and *TRV: GhWRKY70D13* plants at 14 days after inoculation with *V. dahliae*. Each experiment was performed using three independent biological replicates. Differences between groups were compared using the Student's t-test (*P < 0.05; **P < 0.01).

VIGS was employed to knock down the expression of *GhWRKY70D13* and *GhWRKY70D04* in two *V. dahliae* susceptible cultivars, Xinluzao 7 (both genes) and Xincai 7 (only *GhWRKY70D13*). Observation of the expected yellowing leaf phenotype in *TRV: GhCHLI* plants (**Supplementary Figure S3**) and notable reduction of *GhWRKY70D04* and *GhWRKY70D13* in the corresponding VIGS plants (**Supplementary Figures S4A, B**) suggested success of the VIGS experiment. Notably, expression of the corresponding At subgenome homologs of *GhWRKY70D04* and *GhWRKY70D13* was not affected in the corresponding VIGS plants (**Supplementary Figures S4C, D**), suggesting specificity of VIGS. At 14 days after *V. dahliae* inoculation, the control plants (*TRV:00*) and *GhWRKY70D04*-silenced plants displayed severe leaf yellowing and wilting (**Figure 3A** and **Supplementary Figure S5A**). The control plants (*TRV:00*) also showed obvious vascular browning (**Figure 3D**). In contrast, the *GhWRKY70D13*-silenced plants were much healthier and did not show obvious vascular browning (**Figures 3B, D** and **Supplementary Figure S5A**). The *GhWRKY70D13*-silenced plants had significantly (*P* < 0.05) lower disease indices (10.0 ± 2.8 and 33.1 ± 10.4%, respectively) than the control plants (24.2 ± 8.3% and 55.8 ± 8.2%, respectively) at 14 and 21 dpi in *V. dahliae* susceptible cultivar Xincai 7 (**Figure 3C**). In another *V. dahliae* susceptible cultivar Xinluzao 7, the *GhWRKY70D13*-silenced plants (46.1 ± 7.8%) had a significantly (*P* < 0.05) lower disease index than the *TRV:00* plants (62.4 ± 6.0%) (**Supplementary Figure S5B**). Compared to the control plants, the *GhWRKY70D13*-silenced plants accumulated less fungal biomass (down by 0.28- and 0.19-fold in *V. dahliae* susceptible cultivar Xinluzao 7 and Xincai 7, respectively) in their stems and had a lower fungal recovery from the stem sections collected from the inoculated plants (**Figures 3E, F** and **Supplementary Figures S5C, D**). These results were further confirmed in the *V. dahliae* resistant cultivar Zhongzhimian 2 (**Supplementary Figure S6**), suggesting that the mechanism conferring the basal *V. dahliae* resistance in Zhongzhimian 2 does not involve *GhWRKY70D13*.

**Figure 4:**
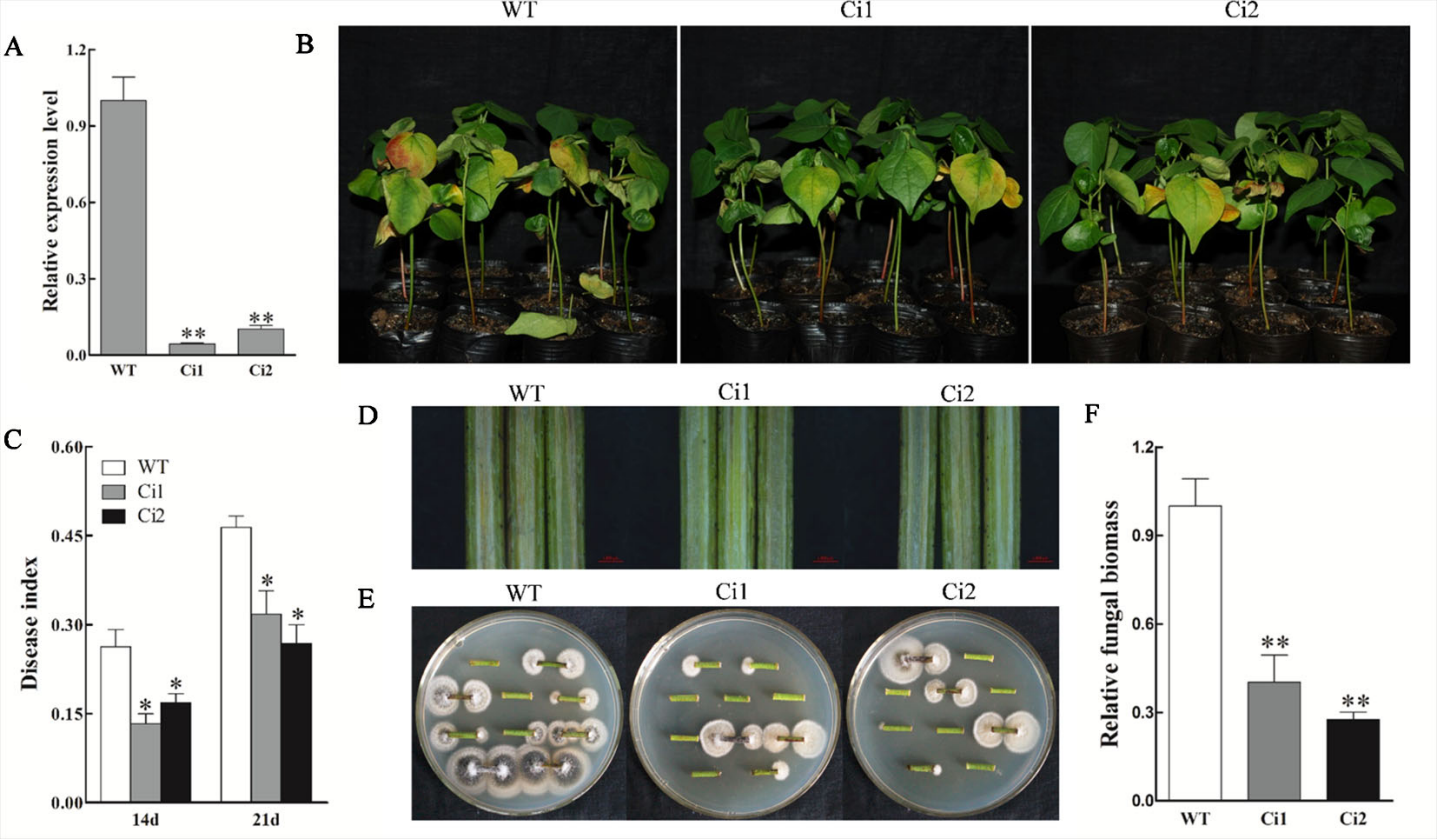
*GhWRKY70D13* RNA interference (RNAi) cotton lines showed enhanced resistance to *Verticillium dahliae* in *V. dahliae*-susceptible cotton cultivar Xincai 7. **(A)** Comparison of the expression level of *GhWRKY70D13* in WT, Ci1, and Ci2 plants. Total RNA was isolated from leaves of two-leaf stage seedlings. *GhUBQ7* was used as the control. **(B)** Disease symptoms of the WT, Ci1, and Ci2 plants after *V. dahliae* inoculation. Photographs were taken at 14 days after inoculation with 30 plants per treatment. **(C)** Disease index of the WT, Ci1, and Ci2 plants at 14 days and 21 days after inoculation with *V. dahliae*. **(D)** Comparison of vascular browning in stems of the WT, Ci1, and Ci2 plants at 14 days after inoculation with *V. dahliae*. **(E)** Comparison of fungal growth in stem sections prepared from the WT, Ci1, and Ci2 plants at 14 days after *V. dahliae* inoculation. Stem sections were plated on potato dextrose agar (PDA) medium. Photographs were taken after 7 days of culture at 25°C. **(F)** Quantitative PCR (qPCR) analysis of the relative fungal biomass in stems from the WT, Ci1, and Ci2 plants at 14 days after inoculation with *V. dahliae*. WT, wild-type; Ci1 and Ci2, two independent *GhWRKY70D13*-RNAi cotton lines. Each experiment was performed using three independent biological replicates. Differences between wild-type and transgenic plants were compared using the Student's t-test (*P < 0.05; **P < 0.01).

To further consolidate the results, we generated stable transgenic cotton lines (in the background of *V. dahliae* susceptible cultivar Xincai 7) with downregulated *GhWRKY70D13* using RNAi and performed disease assay using two T_3_ lines (Ci1 and Ci2). As a result, we confirmed that downregulation of *GhWRKY70D13* was able to enhance cotton's resistance to *V. dahliae* (**Figure 4**).

### Transcriptome Analysis of Wild Type and *GhWRKY70D13*-Ribonucleic Acid Interference Transgenic Line Infected by *Verticillium dahliae*

To explore the effect of downregulation of *GhWRKY70D13* on cotton's response to *V. dahliae* infection, we compared the transcriptome profiles of the *GhWRKY70D13-*RNAi line (Ci, a mixture of Ci1 and Ci2) with those of WT using RNAs isolated from roots of 0, 24, and 72 hpi seedlings. Approximately 40–58 million clean reads per sample were generated (**Supplementary Table S5**). After mapping the clean reads to the *G. hirsutum* reference genome (Wang et al., 2019), we calculated the expression level of each expressed gene based on FPKM and identified DEGs between Ci and WT at each time point.

**Figure 5:**
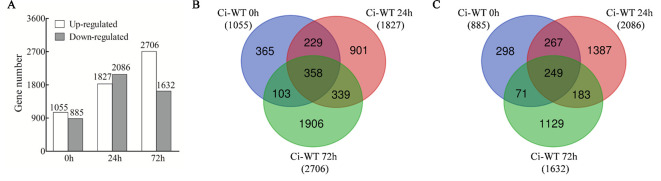
Comparative analysis of differentially expressed genes (DEGs) between the WT and Ci plants following *V. dahliae* inoculation at different time points. **(A)** Number of up-regulated or down-regulated DEGs between WT and Ci at 0, 24, and 72 h after *V. dahliae* inoculation. **(B)** Venn diagram showing up-regulated DEGs at different time points after *V. dahliae* inoculation in Ci compared with WT. **(C)** Venn diagram showing down-regulated DEGs at different time points after *V. dahliae* inoculation in Ci compared with WT. WT, wild-type; Ci, a mixed sample of *GhWRKY70D13-*RNAi line 1 (Ci1) and *GhWRKY70D13-*RNAi line 2 (Ci2).

In total, 4,201 non-redundantly upregulated DEGS and 3,584 non-redundantly downregulated DEGs were identified (**Supplementary Tables S6** and **S7**). The DEGs identified at 0 hpi were results of down-regulation of *GhWRKY70D13*. The DEGs identified at 24 or 72 hpi but not at 0 hpi were potential candidates responding to *V. dahliae* infection. Based on this rationale, down-regulation of *GhWRKY70D13* resulted in 1,055 and 885 up- and down-regulated DEGs, respectively (**Figure 5A**). For the two time points, 1,240 up-regulated and 1,570 down-regulated DEGs were identified at 24 hpi (**Figure 5B**), 2,245 up-regulated and 1,312 down-regulated DEGs were identified at 72 hpi (**Figure 5C**).

### Analysis of Enriched Pathways

To determine what pathways were affected in the roots of Ci and WT plants by *V. dahliae* infection; 3,146 upregulated genes (group I) and 2,699 downregulated genes (group II) due to *V. dahliae* infection were annotated using the KOBAS 2.0 software (**Supplementary Tables S8** and **S9**). Of the top 15 KEGG enriched pathways from each group, 5 were found to be overlapped between the two groups (**Supplementary Figure S7**, **Supplementary Tables S10** and **S11**). These pathways included limonene and pinene degradation, stilbenoid diarylheptanoid and gingerol biosynthesis, biosynthesis of secondary metabolites, alpha-linolenic acid metabolism, and plant hormone signaling transduction. Pathways unique to group II included carotenoid biosynthesis, carbon fixation in photosynthetic organisms, circadian rhythm–plant, cysteine and methionine metabolism, glycolysis/gluconeogenesis, ABC transporters, glucosinolate biosynthesis, brassinosteroid biosynthesis, flavonoid biosynthesis, and ribosome. Several pathways related to plant's response against *V. dahliae* were identified in group I, consistent with results previously reported (Zhang et al., 2013). Plant hormone signaling transduction pathways, including ET and JA, were the most enriched pathways in group I. Therefore, ET and JA signaling pathways might play critical roles in *GhWRKY70D13*-mediated regulation of *V. dahliae* resistance in cotton.

### Silencing of *GhWRKY70D13* Positively Regulates Cotton’s Resistance to *Verticillium dahliae* Through Activation of the Ethylene and Jasmonic Acid Signaling Pathways

**Figure 6:**
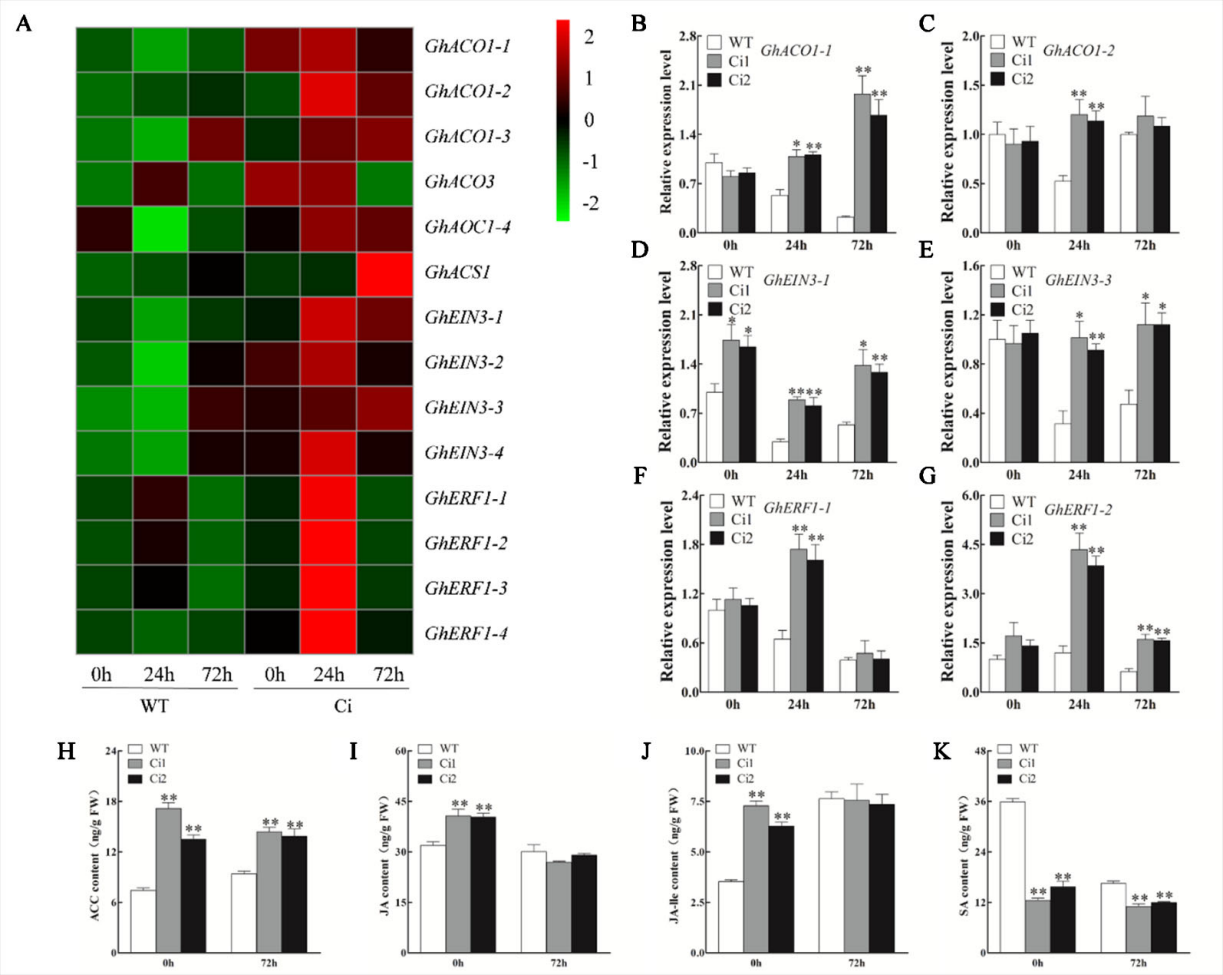
Reverse transcription-quantitative PCR analysis of the expression level of the ethylene (ET) biosynthesis and response genes, and the content of 1-aminocyclopropane-1-carboxylic (ACC), jasmonic acid (JA), JA-isoleucine (JA-Ile), and salicylic acid (SA) in the WT and *GhWRKY70D13-*RNA interference (RNAi) plants inoculated with *Verticillium dahliae*. **(A)** Comparison of the expression patterns of the ET biosynthesis and response genes in the WT and Ci plants infected by *V. dahliae* based on RNA-seq. **(B–G)** Relative expression levels of **(B)** *GhACO1-1*, **(C)** *GhACO1-2*, **(D)** *GhEIN3-1*, **(E)** *GhEIN3-3*, **(F)** *GhERF1-1*, **(G)** *GhERF1-2* at 0, 24, and 72 h after inoculation with *V. dahliae* in the WT, Ci1, and Ci2 plants. **(H–K)** The content of **(H)** ACC, **(I)** JA, **(J)** JA-Ile, and **(K)** SA in the WT, Ci1 and Ci2 plants at 0 h and 72 h after inoculation with *V. dahliae*. WT, wild-type; Ci1 and Ci2, two independent *GhWRKY70D13*-RNAi cotton lines; Ci, a mixed sample of *GhWRKY70D13-*RNAi line 1 (Ci1) and *GhWRKY70D13-*RNAi line 2 (Ci2). Each experiment was performed using three independent biological replicates. Differences between wild-type and transgenic plants were compared using the Student's t-test (*P < 0.05; **P < 0.01).

To investigate the role of *GhWRKY70D13*-mediated regulation of *V. dahliae* resistance through ET and JA signaling, we quantified the expression levels of ET biosynthesis genes (*GhACO1-1*, *GhACO1-2*) and response genes (*GhEIN3-1*, *GhEIN3-2*, *GhERF1-1*, and *GhERF1-2*) in *TRV: 00* and *TRV: GhWRKY70D13* plants following *V. dahliae* infection using RT-qPCR. At 24 and 72 hpi, the expression levels of *GhACO1*, *GhEIN3*, and *GhERF1* were higher in the *TRV: GhWRKY70D13* plants than in the *TRV: 00* plants (**Supplementary Figure S8**). Consistently, transcriptome data showed that numerous ET signaling pathway genes, including *GhACO1*, *GhEIN3*, and *GhERF1*, had an increased expression level in Ci than in WT (**Figure 6A** and **Supplementary Table S12**), and were further induced in both Ci1 and Ci2 at 24 hpi and/or 72 hpi (**Figures 6B–G**). Consequently, the production of endogenous ACC in Ci1 and Ci2 was significantly (*P* < 0.01) increased by 132.1 and 82.2%, respectively, compared to WT at 0 hpi (**Figure 6H**). Ci1 and Ci2 also showed 53.2 and 47.0% increase of the content of endogenous ACC at 72 hpi, respectively (**Figure 6H**). These results suggest that silencing of *GhWRKY70D13* positively regulates the ET-mediated defense response.

**Figure 7:**
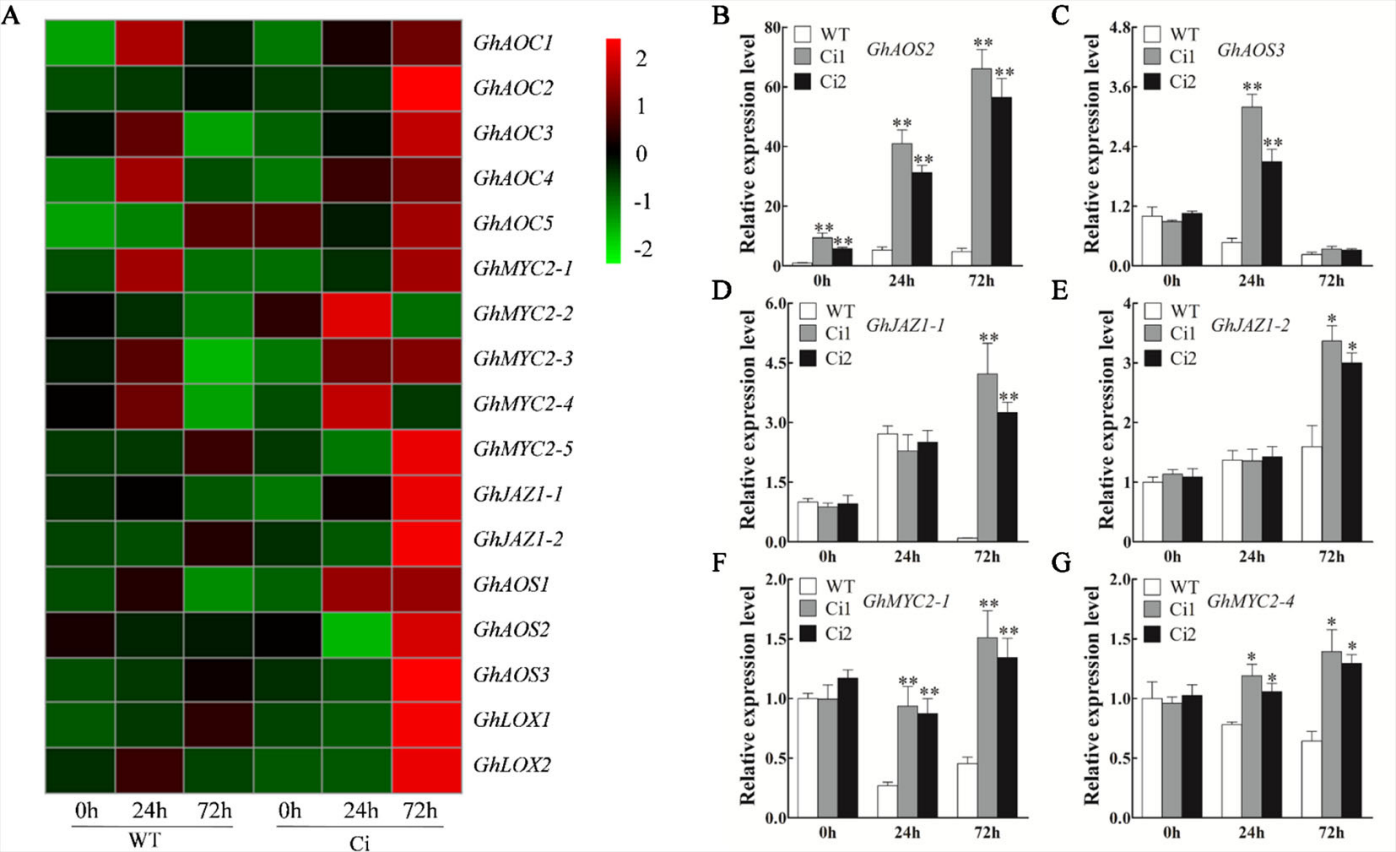
Reverse transcription-quantitative PCR analysis of the expression level of the jasmonic acid (JA) biosynthesis and response genes in the WT and *GhWRKY70D13-*RNAi plants inoculated with *Verticillium dahliae*. **(A)** Comparison of the expression patterns of the JA biosynthesis and response genes in the WT and Ci plants inoculated with *V. dahliae* based on RNA-seq. **(B–G)** RT-qPCR analysis of **(B)** *GhAOS2*, **(C)** *GhAOS3*, **(D)** *GhJAZ1-1*, **(E)** *GhJAZ1-2*, **(F)** *GhMYC2-1*, **(G)** *GhMYC2-4* at 0, 24, and 72 h after inoculation with *V. dahliae* in the WT, Ci1, and Ci2 plants. WT, wild-type; Ci1 and Ci2, two independent *GhWRKY70D13*-RNAi cotton lines; Ci, a mixed sample of *GhWRKY70D13-*RNAi line 1 (Ci1) and *GhWRKY70D13-*RNAi line 2 (Ci2). Each experiment was performed using three independent biological replicates. Differences between wild-type and transgenic plants were compared using the Student's t-test (*P < 0.05; **P < 0.01).

Compared to the *TRV: 00* control plants, the *TRV: GhWRKY70D13* plants showed up-regulation of JA biosynthesis and response genes, such as *GhAOS*, *GhJAZ1*, and *GhMYC2* in response to *V. dahliae* inoculation (**Supplementary Figure S9**). Both RNA-seq and qPCR results showed that JA signaling pathway genes were up-regulated in *GhWRKY70D13-*RNAi lines compared to WT following *V. dahliae* infection (**Figure 7A**, **Supplementary Table S13**, and **Figures 7B–G**). At 0 hpi, the *GhWRKY70D13-*silenced plants Ci1 and Ci2 produced 40.65 ± 3.55 and 40.36 ± 1.89 ng/g JA and 7.28 ± 0.42 and 6.28 ± 0.35 ng/g JA-Ile, respectively, which was significantly (*P* < 0.01) higher than the WT plants (31.88 ± 2.00 ng/g JA and 3.53 ± 0.16 ng/g JA-Ile) (**Figures 6I, J**). However, JA and JA-Ile contents were not significantly different between *GhWRKY70D13-*silenced and WT plants at 72 hpi (**Figures 6I, J**). Similarly, we analyzed the expression levels of SA signaling pathway genes and quantified the SA content in Ci1, Ci2, and WT before and after *V. dahliae* infection. The four potential genes (*GhPAL1*–*GhPAL4*) involved in SA biosynthesis seemed to have different expression patterns in response to down-regulation of *GhWRKY70D13* and *V. dahliae* infection. Similar situation was observed for the SA response genes (**Supplementary Figure S10** and **Supplementary Table S14**). Nevertheless, the content of SA was significantly (*P* < 0.01) lower in Ci1 and Ci2 than in WT at both 0 and 72 hpi (**Figure 6K**).

## Discussion

WRKY proteins play an important role in plant growth and development, as well as in regulating responses to pathogen infections. Numerous studies demonstrated that AtWRKY54 and AtWRKY70 co-operatively modulate plant growth, leaf senescence, drought response, and resistance to pathogens (Besseau et al., 2012; Chen et al., 2017; Li et al., 2017). We identified 10 GhWRKY70s in *G. hirsutum* using AtWRKY70 as a query and found that these GhWRKY70s are also closely related to AtWRKY54 (**Supplementary Figure S1B**), suggesting similar functions of GhWRKY70s as that of AtWRKY70 and AtWRKY54. Most *GhWRKY70* genes had a much higher expression level in vegetative tissues than in reproductive tissues, indicating that those *GhWRKY70* genes may act mainly in vegetative tissues. For example, similar to *AtWRKY70* (Chen et al., 2017), *GhWRKY70A06* and *GhWRKY70D06* were highly expressed in leaves. It would be of interest to know whether they are involved in leaf senescence in cotton. *GhWRKY70D02* and *GhWRKY70D05a* were significantly highly expressed in ovule, implying their important roles in ovule development. WRKY genes exhibited extensive cross-regulation with functional redundancy (Pandey and Somssich, 2009; Rushton et al., 2010). Similar expression patterns of the *GhWRKY70* homologs, particularly the At and Dt homologs of each *GhWRKY70*, suggest their possible redundant functions. Analysis of gene expression pattern provides important clues of gene functions (Xue et al., 2008). Cis-regulatory elements found at the gene promoter regions directly influence gene expression and hence control plant development and responses to environmental stress (Wittkopp and Kalay, 2011; Puranik et al., 2012). Consistent with the presence of at least one hormone-responsive element in the promoters of the 10 *GhWRKY70* genes (**Supplementary Figure S2**), most *GhWRKY70* genes responded to SA, JA, and/or ETH treatment (**Figures 2A–C**).

Several studies have reported the involvement of WRKY70 in regulation of disease resistance. For example, *Arabidopsis wrky70* mutant showed decreased response to biotroph *Erysiphe cichoracearum* infection (Li et al., 2004; Li et al., 2006). In wheat, *TaWRKY70*-silenced plants displayed reduced tolerance to *Puccinia striiformis* f. sp. *tritici* infection (Wang et al., 2017). Overexpression of *PsnWRKY70* increased resistance to the leaf blight disease in transgenic *Populus simonii* × *Populus nigra* plants (Zhao et al., 2017). Our previous study found that *GhWRKY70A05a* (*Gh_A05G2378*) negatively regulates the defense response against *V. dahliae* (Xiong et al., 2019). Up-regulation of *GhWRKY70A05a* in *V. dahliae* resistant cotton cultivar and down-regulation of the gene in *V. dahliae* susceptible cotton cultivar suggest that *GhWRKY70A05a* is a positive regulator of a negative regulator of *V. dahliae*-resistance. Herein, two other *GhWRKY70* genes, *GhWRKY70D04* and *GhWRKY70D13,* with different expression patterns were silenced by VIGS to investigate their responses to *V. dahliae* infection. Silencing of *GhWRKY70D13* but not *GhWRKY70D04* resulted in improved tolerance to *V. dahliae* (**Supplementary Figure S5**). The role of *GhWRKY70D13* in disease response was further confirmed in the stable transgenic RNAi cotton plants (**Figure 4**). These findings indicated that *GhWRKY70D13* played a negative role in defense against *V. dahliae* in cotton. Consistent with this finding, we found that the expression levels of *GhWRKY70D13* were much higher in the *V. dahliae*-susceptible cultivar Xinluzao 7 than in the *V. dahliae*-resistant cultivar Zhongzhimian 2 at 12 and 24 hpi (**Figure 2D**). V991 is an aggressive defoliating strain, it's necessary to use another nondefoliating strain to have a comprehensive understanding on the role of *GhWRKY70D13* related to *V. dahliae* resistance. In *Arabidopsis*, *AtWRKY70* regulates the susceptibility to necrotrophic *Alternaria brassicicol* (Li et al., 2004; Li et al., 2006). ET and JA signaling pathways are the major pathways involved in defense against necrotrophic pathogens (Glazebrook, 2005). *AtWRKY70* was induced by SA and JA as well as by infection of several pathogens (Kalde et al., 2003; Li et al., 2004). In wheat, the expression of *TaWRKY70* is increased upon exogenous application of SA and ET, but down-regulated by MeJA stimulation (Wang et al., 2017).

ACO facilitates ET biosynthesis in plant tissues (Nakatsuka et al., 1998; Ruduś et al., 2013). EIN3 and ERF1 are linked to the ET signaling pathway and have been implicated in regulating plant immune responses (Nie et al., 2012; Meng et al., 2013). Increasing evidence suggested that ET-associated genes regulate cotton resistance to *V. dahliae*. Cotton *ACO1*, *ACO3*, and *ERF1* genes were activated upon *V. dahliae* infection (Xu et al., 2011). Overexpressing *GhERF1-like* in cotton enhanced resistance to *V. dahliae* (Guo et al., 2016). Knockdown of *GhERF6* decreased cotton's tolerance to *V. dahliae* infection (Yang et al., 2015). We found that the expression levels of *GhACO1*, *GhEIN3*, and *GhERF1* were increased in the roots of Ci1, Ci2, and *TRV*: *GhWRKY70D13* plants (**Figures 6A–G** and **Supplementary Figure S8**). *GhWRKY70D13*-RNAi plant had a significantly increased level of ACC content compared to WT at both before and after *V. dahliae* inoculation (**Figure 6H**). These results demonstrated that *GhWRKY70D13* negatively regulated the ET signaling pathway that positively regulates defense against *V. dahliae* infection in cotton.

Numerous studies have shown that ET and JA synergistically regulate plant tolerance to necrotrophic pathogens (Wang et al., 2015; Li et al., 2018). Our results showed that some JA biosynthesis genes and response genes were significantly up-regulated in *TRV*: *GhWRKY70D13* plants after *V. dahliae* inoculation compared with *TRV*: *00* (**Supplementary Figure S9**). Consistently, the expression levels of *GhAOS*, *GhJAZ1*, and *GhMYC2* were also up-regulated in the *GhWRKY70D13-*RNAi plant (**Figure 7**). Thus, silencing of *GhWRKY70D13* might positively regulate the JA signaling pathway to increase cotton's resistance to *V. dahliae*. Our findings consist with those reported previously (Gao et al., 2013; Zhang et al., 2013). Suppression of *GbWRKY1* and *GhCYP94C1* increased the expression of JA-associated genes and improved resistance to *V. dahliae* (Li et al., 2014a; Sun et al., 2014). In contrast, *GbSSI2*-silenced cotton plants displayed decreased resistance to *V. dahliae* due to suppressed JA accumulation and signaling (Gao et al., 2013). Recently, Yang et al. reported that overexpression of JAZ inhibited JA signaling (Yang et al., 2012). Similar results were found in cotton, in which *GhJAZ2* acted as a repressor of JA signaling and negatively regulated the resistance of cotton to *V. dahliae* (He et al., 2018). We found that the contents of JA and JA-Ile were significantly higher in the *GhWRKY70D13*-silenced plant (Ci1 and Ci2) than in WT but such difference vanished at 72 hpi (**Figures 6I, J**), which might be related to the expression patterns of the two *GhJAZ1* genes that had a similar level in Ci1, Ci2, and WT uninfected by *V. dahliae* but were significantly up-regulated in Ci1 and Ci2 at 72 hpi (**Figures 7D, E**).

Studies in *Arabidopsis* showed that WRKY70 is a negative regulator of SA biosynthesis, but a positive regulator of SA-responsiveness (Wang et al., 2006). Knock-down the expression level of *GhWRKY70D13 *seemed to have different effect on SA biosynthesis and response genes in cotton (**Supplementary Figure S10**). Compared with WT, Ci1 and Ci2 had a significantly decreased SA content at both before and after *V. dahliae* infection, although the SA content in WT was dramatically reduced due to *V. dahliae* infection (**Figure 6K**). It is of interest to know whether this is a result of the antagonistic relationship between the JA and SA signaling pathways (Bari and Jones, 2009; Thaler et al., 2012). On the other hand, we showed previously that suppression of *GhWRKY70A05a* expression improved resistance to *V. dahliae* by down-regulating SA signaling pathways and up-regulating JA signaling pathways (Xiong et al., 2019). This is different to the function of *GhWRKY70D13* which seems to negatively regulate ET and JA signaling pathways.

**Figure 8:**
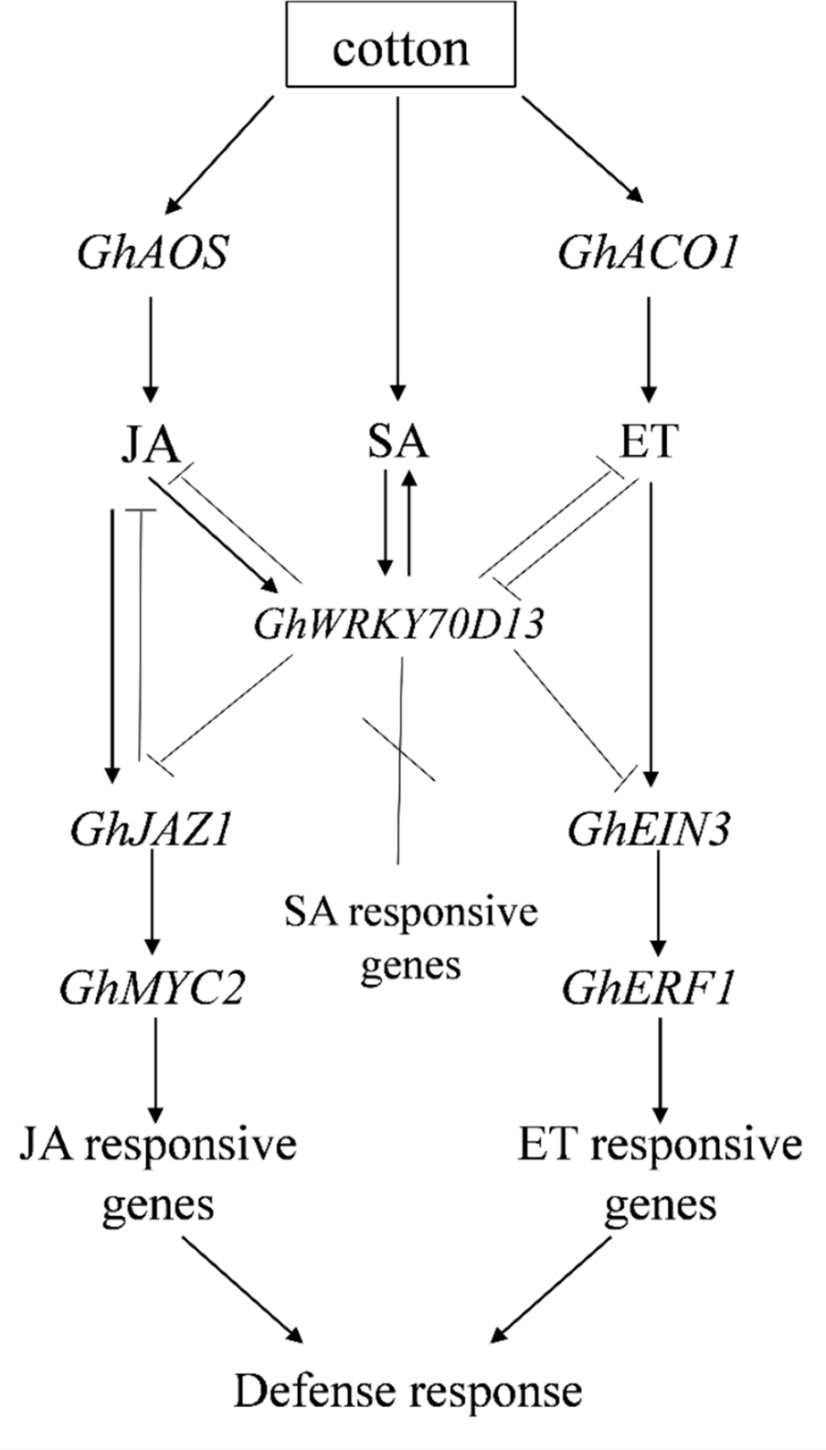
A working model describing the regulatory role of *GhWRKY70D13* on salicylic acid (SA), jasmonic acid (JA), and ethylene (ET) signaling in cotton upon infection of *Verticillium dahliae*.

In summary, we showed that down-regulation of *GhWRKY70D13* in cotton significantly enhanced accumulation of ACC, JA, and JA-Ile, and impaired SA biosynthesis, consequently enhanced cotton's resistance to *V. dahliae*. *GhWRKY70D13* negatively regulates cotton's response to *V. dahliae* infection by downregulating the ET and JA signaling pathways (**Figure 8**). The role of cross-talk between the JA and ET signaling pathways in cotton's resistance to *V. dahliae* requires however further investigations.

## Data Availability Statement

All sequencing data generated in this study can be found in the NCBI using accession number PRJNA578842.

## Author Contributions

JS and FX conceived and designed the experiments. X-PX conducted most of the experiments, analyzed the data and wrote the manuscript. S-CS and X-YZ provided technical assistance to X-PX. X-YZ, Y-JL, and FL contributed reagent and materials. Q-HZ and JS revised the manuscript. All authors reviewed and approved the final manuscript.

## Funding

This work was supported by the National Key Research and Development Program of China (No. 2016YFD0100200, 2016YFD0101900) and the Genetically Modified Organisms Breeding Major Project of China (Grant No. 2016ZX08005-005).

## Conflict of Interest

The authors declare that the research was conducted in the absence of any commercial or financial relationships that could be construed as a potential conflict of interest.

## Supplementary Material

The Supplementary Material for this article can be found online at: https://www.frontiersin.org/articles/10.3389/fpls.2020.00069/full#supplementary-material
