# Corrigendum: *GhWRKY70D13* Regulates Resistance to *Verticillium* *dahliae* in Cotton Through the Ethylene and Jasmonic Acid Signaling Pathways

**DOI:** 10.3389/fpls.2020.01045

**Published:** 2020-07-10

**Authors:** Xian-Peng Xiong, Shi-Chao Sun, Xin-Yu Zhang, Yan-Jun Li, Feng Liu, Qian-Hao Zhu, Fei Xue, Jie Sun

**Affiliations:** ^1^ Key Laboratory of Oasis Eco-agriculture, College of Agriculture, Shihezi University, Shihezi, China; ^2^ Agriculture and Food, CSIRO, Canberra, ACT, Australia

**Keywords:** *Gossypium hirsutum*, *Verticillium dahliae*, *GhWRKY70D13*, ribonucleic acid interference, jasmonic acid, ethylene

## Incorrect Author Name

An author name was incorrectly spelled as **Xiang-Peng Xiong**. The correct spelling is **Xian-Peng Xiong**.

The authors apologize for this error and state that this does not change the scientific conclusions of the article in any way. The original article has been updated.

