# Supplementary material for: GhWRKY70D13 Regulates Resistance to Verticillium dahliae in Cotton Through the Ethylene and Jasmonic Acid Signaling Pathways
Source: Front Plant Sci. 2020 Feb 25;11:69. doi: 10.3389/fpls.2020.00069 (PMC7052014; doi:10.3389/fpls.2020.00069)

Figure S1 Sequence alignment and phylogenetic analysis of *Gossypium WRKY70* genes with their homologs from *Arabidopsis*.

(A) Alignment of the WRKY domain from *WRKY70* genes of *G. hirsutum*, *G. arboreum* and *G. raimondii*. The protein sequences were aligned using ClustalX 1.83. (B) Phylogenetic relationship analysis of the group III *WRKY* genes in *Arabidopsis thaliana* and the *WRKY70* genes in *G. raimondii*, *G. arboreum* and *G. hirsutum*. The phylogenetic tree was constructed using amino acid sequences and the neighbor-joining (NJ) method with 1000 bootstrap replicates in MEGA 7.0. At, *A. thaliana*; Gr, *G. raimondii*; Ga, *G. arboreum*; Gh, *G. hirsutum*.

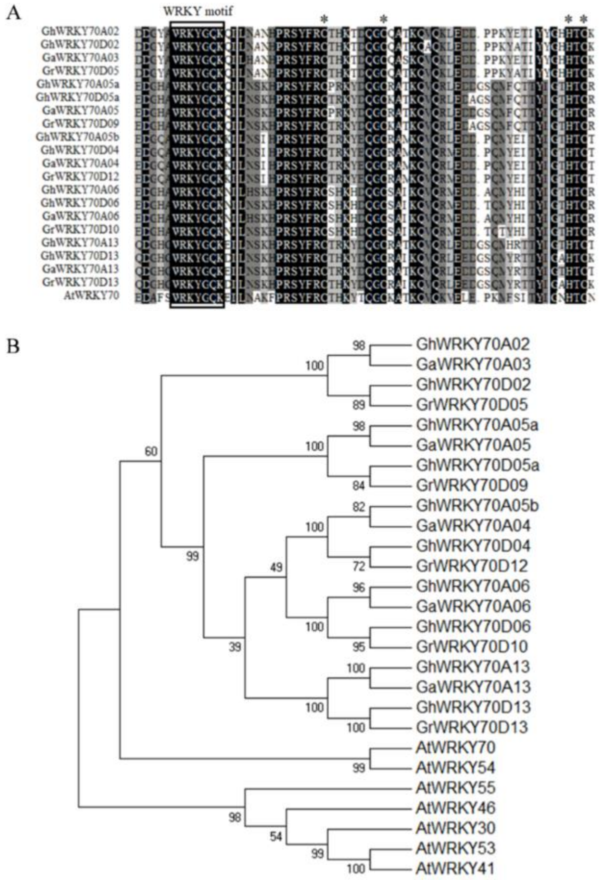

Figure S2 Analysis of cis-elements in the promoters of *GhWRKY70* genes. The cis-elements response to different hormones are presented as bar graphs with different colors.

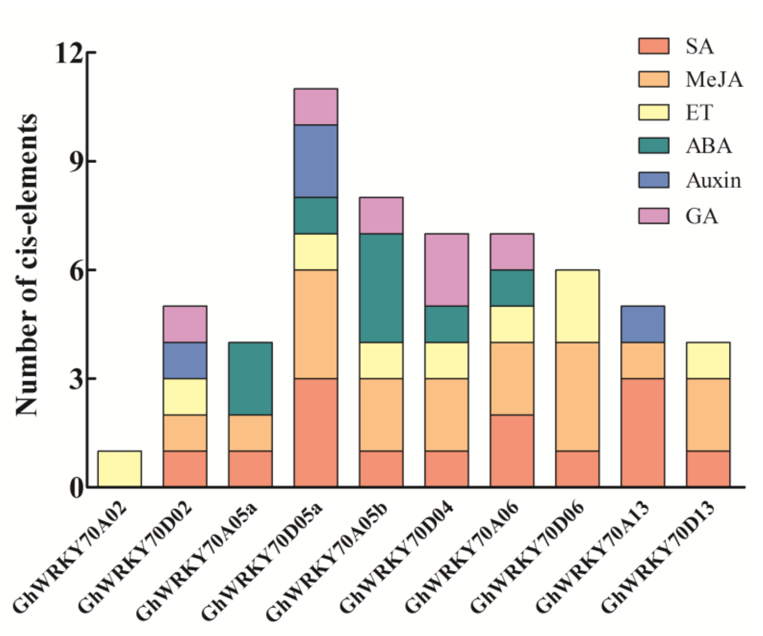

Figure S3 Phenotypes of the cotton plants two weeks after infiltration with *TRV: GhCHLI*. Silencing of *GhCHLI* caused leaf yellowing in (A) Xincai 7 (*V. dahliae*-susceptible), (B) Xinluzao 7 (*V. dahliae*-susceptible) and (C) Zhongzhimian 2 (*V. dahliae*-resistant).

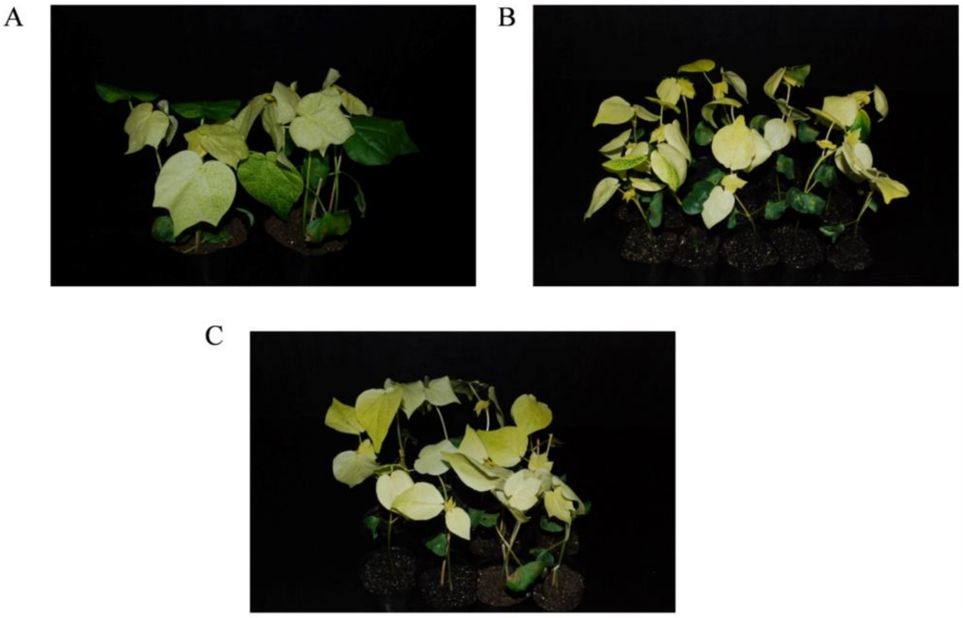

Figure S4 Analysis of the expression levels of the VIGS target genes.

Comparison of *GhWRKY70D04* (A) and *GhWRKY70A05b* (B) in *TRV:00* and *TRV: GhWRKY70D04* cotton plants.

Comparison of *GhWRKY70D13* (C) and *GhWRKY70A13* (D) in *TRV:00* and *TRV: GhWRKY70D13* cotton plants. Total RNA was isolated from leaves at 10 days post agroinfiltration. *GhUBQ7* was used as the control. Each experiment was performed using three independent biological replicates. Differences between groups were compared using the Student's t-test (* $P < 0.05$; ** $P < 0.01$).

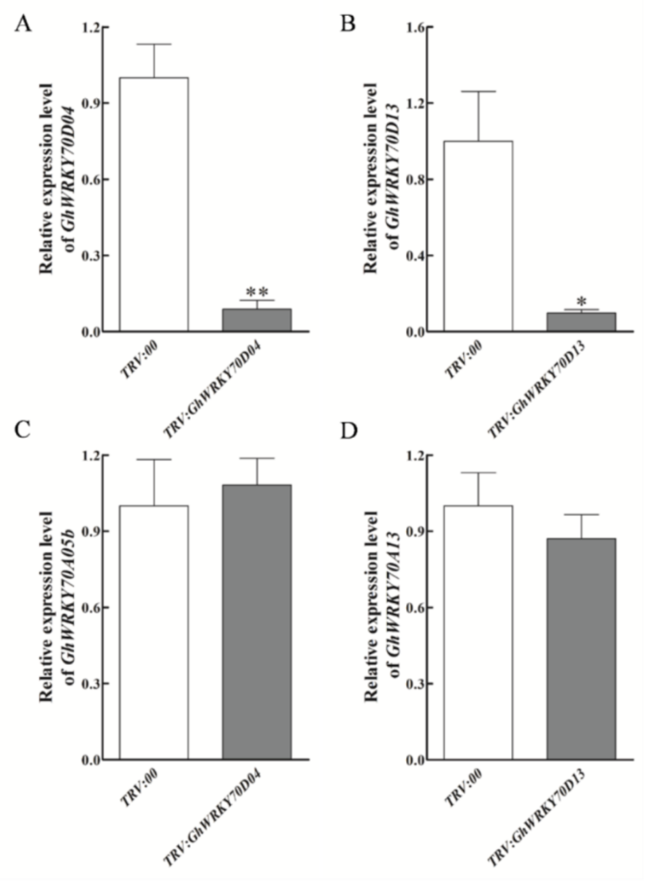

Figure S5 Silencing of *GhWRKY70D13* increased resistance to *V. dahliae* in *V. dahliae*-susceptible cotton cultivar Xinluzao 7.

(A) Disease symptoms of the *TRV:00*, *TRV: GhWRKY70D04* and *TRV: GhWRKY70D13* cotton plants after *V. dahliae* inoculation. Photographs were taken at 14 days after inoculation with 30 plants per treatment. (B) Disease index of the *TRV:00*, *TRV: GhWRKY70D04* and *TRV: GhWRKY70D13* plants at 14 days and 21 days after inoculation with *V. dahliae*. Each experiment was performed using three independent biological replicates. (C) Comparison of fungal growth in stem sections prepared from the *TRV:00*, *TRV: GhWRKY70D04* and *TRV: GhWRKY70D13* plants at 14 days after *V. dahliae* inoculation. Stem sections were plated on PDA medium. Photographs were taken after 7 days of culture at 25 ℃. (D) Relative fungal biomass in stems of the *TRV:00, TRV: GhWRKY70D04* and *TRV: GhWRKY70D13* plants at 14 days after inoculation with *V. dahliae* . qPCR was used in the analysis. Each experiment was performed using three independent biological replicates. Differences between groups were compared using the Student's t-test (* $P < 0.05$; ** $P < 0.01$).

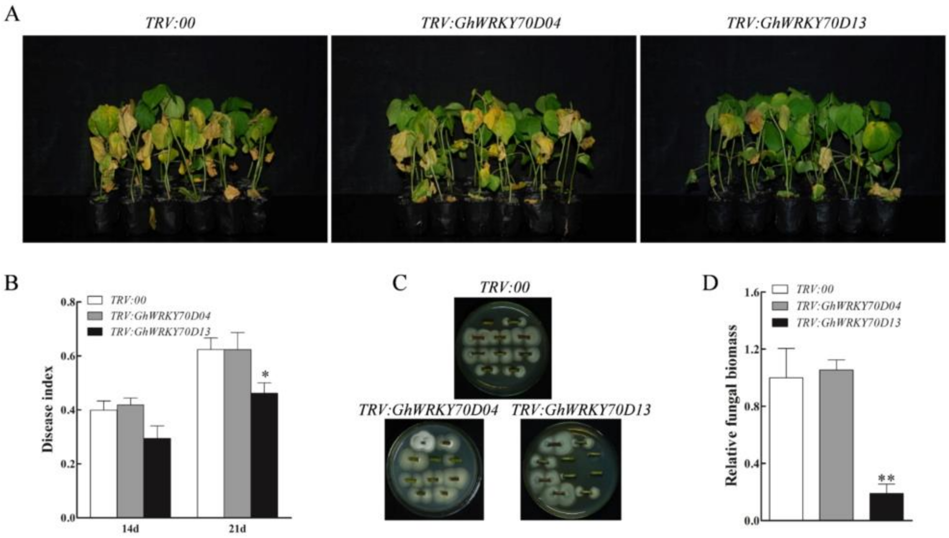

Figure S6 Silencing of *GhWRKY70D13* enhanced resistance to *V. dahliae* in *V. dahliae*-resistant cotton cultivar Zhongzhimian 2.

(A) The expression level of *GhWRKY70D13* in the *TRV:00* and *TRV: GhWRKY70D13* plants. Total RNA was isolated from leaves at 10 days post agroinfiltration. *GhUBQ7* was used as the control. (B) Disease symptoms of the *TRV:00* and *TRV: GhWRKY70D13* plants after *V. dahliae* inoculation. Photographs were taken at 14 days after inoculation with 30 plants per treatment. (C) Disease index of the *TRV:00* and *TRV: GhWRKY70D13* plants at 14 days and 21 days after inoculation with *V. dahliae* .

(D) Comparison of vascular browning in stems of the *TRV:00* and *TRV: GhWRKY70D13* plants at 14 days after inoculation with *V. dahliae*. (E) Comparison of fungal growth in stem sections prepared from the *TRV:00* and *TRV: GhWRKY70D13* plants at 14 days after *V. dahliae* inoculation. Stem sections were plated on PDA medium. Photographs were taken after 7 days of culture at 25 ℃. (F) qPCR analysis of the relative fungal biomass in stems of the *TRV:00* and *TRV: GhWRKY70D13* plants at 14 days after inoculation with *V. dahliae*. Each experiment was performed using three independent biological replicates. Differences between groups were compared using the Student's t-test (* $P < 0.05$; ** $P < 0.01$).

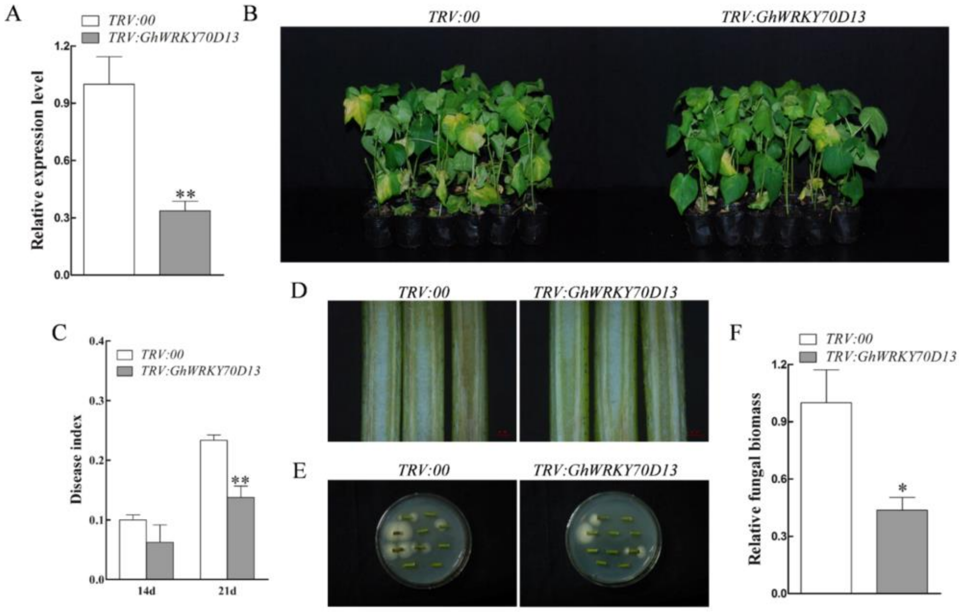

Figure S7 KEGG analysis of all up-regulated (3146) or down-regulated (2699) differentially expressed genes (DEGs) after *V. dahliae* inoculation in Ci1 compared with WT. WT, wild-type; Ci, *GhWRKY70D13*-RNAi line Ci1 and *GhWRKY70D13*-RNAi line Ci2 mixed.

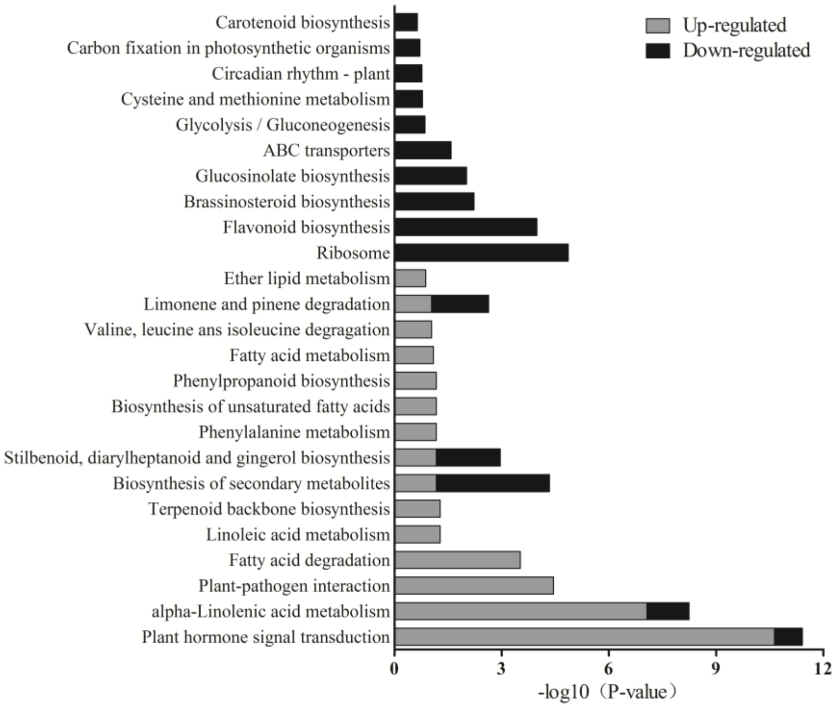

Figure S8 Reverse transcription-quantitative PCR analysis of ET biosynthesis and response genes in the *TRV:00* and *TRV:GhWRKY70D13* plants at 0 h, 24 h and 72 h after inoculation with *V. dahliae*. Each experiment was performed using three independent biological replicates. Differences between groups were compared using the Student's t-test (* $P < 0.05$; ** $P < 0.01$).

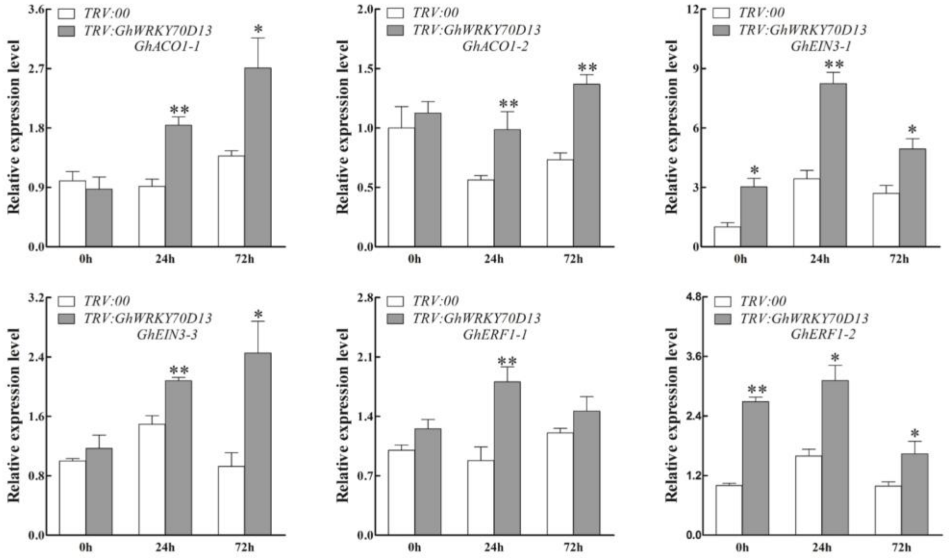

Figure S9 Reverse transcription-quantitative PCR analysis of JA biosynthesis and response genes in the *TRV:00* and *TRV:GhWRKY70D13* plants at 0 h, 24 h and 72 h after inoculation with *V. dahliae*. Each experiment was performed using three independent biological replicates. Differences between groups were compared using the Student's t-test (* $P < 0.05$; ** $P < 0.01$).

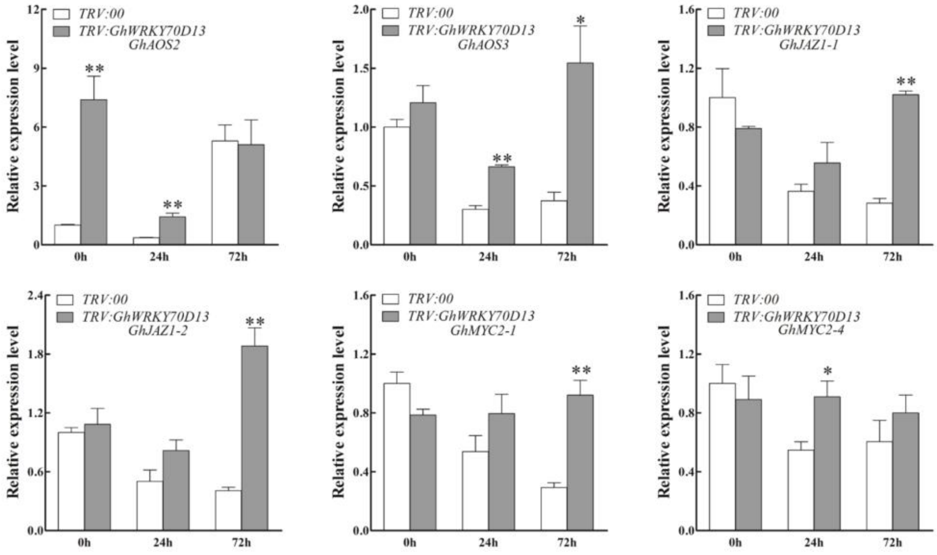

Figure S10 Expression analysis of salicylic acid biosynthesis and response genes using RNA-seq.

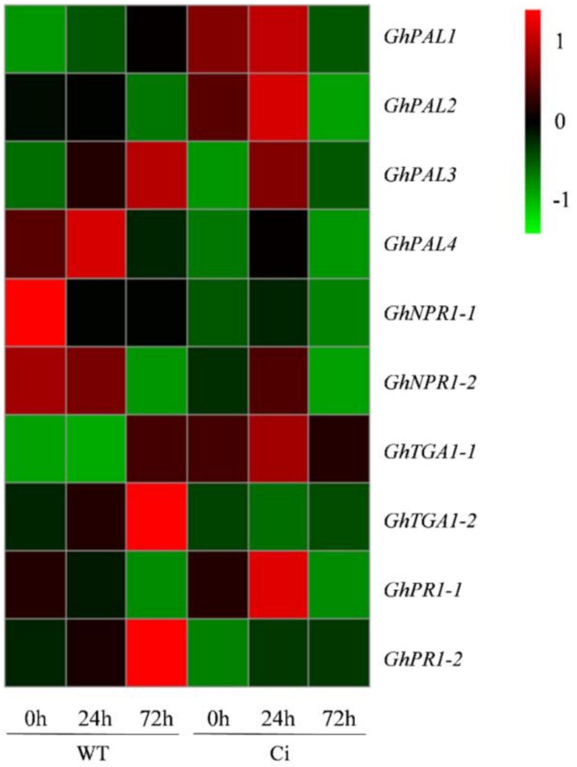

Supplement: Supplementary file 1 [file DataSheet_1.pdf]
